# Nutritional Impact on Metabolic Homeostasis and Brain Health

**DOI:** 10.3389/fnins.2021.767405

**Published:** 2022-01-27

**Authors:** Lionel Carneiro, Luc Pellerin

**Affiliations:** ^1^Department of Biological Chemistry and Pharmacology, Ohio State University, Columbus, OH, United States; ^2^Inserm U1082, Université de Poitiers and CHU de Poitiers, Poitiers, France

**Keywords:** nutrition, aging, neurological disorder, nutrient sensing, cognition, metabolism

## Abstract

Aging in modern societies is often associated with various diseases including metabolic and neurodegenerative disorders. In recent years, researchers have shown that both dysfunctions are related to each other. Although the relationship is not fully understood, recent evidence indicate that metabolic control plays a determinant role in neural defects onset. Indeed, energy balance dysregulation affects neuroenergetics by altering energy supply and thus neuronal activity. Consistently, different diets to help control body weight, blood glucose or insulin sensitivity are also effective in improving neurodegenerative disorders, dampening symptoms, or decreasing the risk of disease onset. Moreover, adapted nutritional recommendations improve learning, memory, and mood in healthy subjects as well. Interestingly, adjusted carbohydrate content of meals is the most efficient for both brain function and metabolic regulation improvement. Notably, documented neurological disorders impacted by specific diets suggest that the processes involved are inflammation, mitochondrial function and redox balance as well as ATP production. Interestingly, processes involving inflammation, mitochondrial function and redox balance as well as ATP production are also described in brain regulation of energy homeostasis. Therefore, it is likely that changes in brain function induced by diets can affect brain control of energy homeostasis and other brain functions such as memory, anxiety, social behavior, or motor skills. Moreover, a defect in energy supply could participate to the development of neurodegenerative disorders. Among the possible processes involved, the role of ketone bodies metabolism, neurogenesis and synaptic plasticity, oxidative stress and inflammation or epigenetic regulations as well as gut-brain axis and SCFA have been proposed in the literature. Therefore, the goal of this review is to provide hints about how nutritional studies could help to better understand the tight relationship between metabolic balance, brain activity and aging. Altogether, diets that help maintaining a metabolic balance could be key to both maintain energy homeostasis and prevent neurological disorders, thus contributing to promote healthy aging.

## Introduction

Modern societies experience a surge in their aging population due to improved medicine that led to extended life expectancy. However, in parallel with aging of the population, age-related disorders are also on the rise (Thane, 2013; Vilić, 2017). Those age-related disorders include notably metabolic and neurodegenerative disorders as well as cancer (Finkel et al., 2007; Wyss-Coray, 2016; Meldrum et al., 2017). Interestingly, recent research has demonstrated the key role of metabolic regulations on both neurological disorders and cancer. Moreover, the aging process appears to be affected by metabolic control (Yates et al., 2012; World Health Organization [WHO], 2015; Luengo et al., 2017). Therefore, diet is a key element in healthy living and healthy diet as defined by WHO^1^ is of great importance for global health. Hence, it is now admitted that healthy aging can be promoted by a balanced energy homeostasis that can be defined by an equilibrium between energy intake and energy expenditures that allows body weight and internal parameters (glycemia, temperature, pH) to remain constant. In support of this concept, different types of diet have been linked to healthy living. For instance, Japanese and Mediterranean diets are found in populations known for their lower metabolic disorders’ incidence or a higher life expectancy (Martinez-Gonzalez and Martin-Calvo, 2016; Poli et al., 2019; Tomata et al., 2019). Noteworthily, these benefits are well known examples described not only in scientific journals but emerging also among the lay public communication media. Other diet changes for lipid, carbohydrates or protein content or caloric supply, or special diets s (e.g., ketopgenic diet, medium Chain triglycerides diet, caloric restrictions, intermittent fasting…) have been developed to prevent or treat certain disorders including cancer, as well as metabolic or neurodegenerative disorders (Amine et al., 2003; World Health Organization [WHO], 2003; Moynihan and Petersen, 2004; Gano et al., 2014; D’Alessandro et al., 2016; Billingsley et al., 2018; Duan et al., 2018; Reddavide et al., 2018; Klement and Pazienza, 2019; Mittelman, 2020). Ketogenic diet (KD), low glycemic index diet (LGI), or adjusted diets (for polyunsaturated fatty acids, medium chain triglycerides or other, proteins…) are the most documented (Apekey et al., 2009; Kanoski and Davidson, 2011; Paoli, 2014; Sanders, 2014; Vergati et al., 2017; Abdelhamid et al., 2018; Dohrmann et al., 2019; Włodarek, 2019; Carneiro and Leloup, 2020; van Name et al., 2020). However, little is known about the mechanisms involved in the beneficial effects of diets on health. Of note, neurological disorders are often targeted by diet interventions. Therefore, diet utilization in neurological conditions has led to numerous publications on the putative role of diets. Such an observation is of interest since the brain is the highest energy consumer of the body. Indeed, at least 20% of the total body glucose consumption at rest occurs in the brain for its normal activity. In addition, the brain can rapidly adjust energy supply to keep a constant level of nutrient availability (Peters, 2011; Pellerin and Magistretti, 2012). Overall, it appears clear that brain activity and energy homeostasis of the body are tightly linked. Hence, brain 60 disorders are associated with dysregulated energy balance. In addition, obesity or diabetes have been 61 shown to contribute to brain disorders development (Murakami et al., 2010; Val-Laillet et al., 2011; Castanon et al., 2015; Bogdanov et al., 2020; Bremner et al., 2020; Tanaka et al., 2020). In fact, neurological disorders studies provided interesting mechanistic hypotheses thanks to the disease mechanisms knowledge (Gómez-Pinilla, 2008; Gano et al., 2014; Li et al., 2017, 2020; Williams and Cervenka, 2017; Gomez-Pinilla et al., 2021). These observations reinforced the relationship between aging and associated disorders with metabolic regulation. Furthermore, it also indicates that metabolic balance is a key component of healthy aging. It is worth to note that the hypothesized mechanisms involved in the beneficial effect of diets on brain function have also been described in the brain control of energy homeostasis (Carneiro and Leloup, 2020). Therefore, it is likely that disturbed energy balance will contribute to dysfunction of brain control of energy homeostasis. In turn, this dysregulated brain energy homeostasis control would alter brain metabolism and participate to brain disorders. Hence, it is important to fully understand the mechanisms involved in dietary beneficial effects on brain activity to better understand brain control of energy homeostasis as well as brain function and diseases.

In this review, we thus aim to summarize the recent knowledge about the impact of diet on neurological function and disorders. The putative mechanisms at play will be presented, and the relationships with brain regulation of energy homeostasis will be discussed.

## Diet and Brain Function of Healthy Populations

Cognitive responses are dependent on adequate brain function. Exposure to a high fat diet (HFD) has been described to decrease cognitive performances in various tests probing different aspects including working and spatial memory, mood, or spatial memory (Cordner and Tamashiro, 2015). Similarly, a high sucrose diet has also negative impact on cognitive responses (Davis et al., 2020). Therefore, these studies have permitted to demonstrate the deleterious effect of non-healthy diets on cognition. Altogether, obesogenic/diabetogenic diets induce a negative alteration of cognitive functions. However, it remains difficult to discriminate the cognitive decline from obesity or diabetes development. Therefore, to determine the impact of a diet on cognitive function independently of any other metabolic effect, it is necessary to study first the impact of healthy diets on healthy populations.

Among the so-called healthy diets, the Mediterranean diet is described to prevent cognitive decline during aging (Safouris et al., 2015; Aridi et al., 2017). However, these studies only report a decreased risk of developing dementia, and thus a decline in cognitive functions (Safouris et al., 2015; Charisis et al., 2021). In contrast, little is known of the effect of a healthy diet in young healthy people and their cognitive functions. In fact, attempts to determine the impact of a Mediterranean diet on young adult’s cognitive performances led to interesting results (McMillan et al., 2011). In their study, McMillan et al. (2011), gave a specific diet regimen to one group while the control group had no diet changes from their habits. In their study, the authors report an improvement in reaction time during a spatial memory test as well as an increase of accuracy scores. However, other cognitive tests did not show any change after the Mediterranean diet. This restricted impact makes it difficult to conclude on a clear effect of the diet on cognitive function. One possible pitfall is the duration of diet exposure in the study of McMillan et al. (2011). Indeed, in this study, the Mediterranean diet was given for a short period of 10 days. Therefore, a longer term follow up must be done to clearly address the impact of the Mediterranean diet on cognition of healthy adults. In addition, in their study, McMillan et al. (2011) observed a significant improvement of mood. This strong effect following short-term exposure to a diet in a relatively small group of subjects supports the idea of a potentially larger impact of healthy diets on brain function. Altogether, while encouraging, this study needs to be pursued with a larger group and on a longer period. It would be also of interest to compare different dietary habits to identify the factors involved in the putative improvements. Therefore, retrospective and intervention studies could help to determine specific diet composition patterns. Prospective studies would also help to determine chronic effects of diet on health during aging. Indeed, healthy aging could be defined as aging without loss of autonomy due to a disease as oppose to unhealthy aging when a disorder development leads to a loss of independence due to treatments and the need of assistance. Indeed, it is unlikely that aging with complete absence of disease could be considered. However, it is worth to note that healthy aging according to such a definition would be hard to achieve. Nevertheless, a delay in the onset of certain diseases would still help people live longer in healthy conditions. In addition to clearly address the diet effect and test the putative role of specific nutrients or foods, an experimentally controlled diet should be provided to both control and treated groups. Therefore, intervention studies are also necessary.

In another study, the Christian Orthodox Church (COC) fasting diet led to significant results on health and cognition. This specific diet implies a limited intake of animal-based products and a significant increase in fruit and vegetables. Such a nutrient composition makes the diet enriched in fibers and folates while the amount of saturated fatty acids is significantly lowered (Rodopaios et al., 2019). Spanaki et al. (2021) evaluated the cognitive profile of people following the COC fasting diet *vs* a group not adhering to it. The authors reported decreased levels of anxiety and depression and better cognitive performances in the COC fasting diet group. This result supports a beneficial effect of a healthy diet on healthy populations. Indeed, the COC fasting diet is an isocaloric diet, derived from the Mediterranean diet. In addition to prioritizing healthy nutrients (unsaturated fatty acids, complex sugars, fibers…), the COC fasting diet is also classified as an intermittent fasting. Intermittent fasting has been described for its potent impact on metabolic improvements and global health benefits (Mattson et al., 2017, 2018; Mattson, 2019). The study of Spanaki et al. (2021) on COC fasting diet resumes the intermittent fasting benefits while also providing evidence for a cognitive improvement even in healthy people.

On the other hand, other studies of intermittent fasting habits failed to report any positive role on cognition. Thus, studies of individuals that follow Ramadan practices (a fasting during daylight period followed during 28 consecutive days with no food or drink consumption) do not report cognitive improvement although sleep and arousal are positively affected (Dolu et al., 2007; Tian et al., 2011; Meo and Hassan, 2015; Chamari et al., 2016). Nevertheless, few of the studies related to Ramadan fasting focused on cognitive function including memory, attention, or test accuracy for instance. In addition, it is worth to note that the population studied often includes athletes and thus, the effect of exercise and training could mask the impact of diet. Finally, it is important to note that this religious practice of intermittent fasting only lasts one month. Hence, it is possible that significant effects on cognition would only appear after a longer period. Indeed, other intermittent fasting studies show that neurogenesis and brain plasticity, two mechanisms highly involved in cognition, are significantly affected. However, it is important to recall that studies on healthy populations are rare, which makes it difficult to clearly conclude on intermittent fasting and cognition interaction.

In fact, there is very few studies really testing cognitive function in relation with the diet in healthy adults. However, schoolchildren are a healthy population whose cognitive function has been tested in relation with the diet. Interestingly, in these schoolchildren, the diet appears to significantly affect cognitive results (Naveed et al., 2020). Several studies in adolescents show a positive relationship between a low glycemic index breakfast and learning, attention, stress, and even mood (Micha et al., 2010, 2011; Cooper et al., 2011, 2012, 2015; Edefonti et al., 2014). Noteworthily, when compared to adolescents without breakfast, both low and high glycemic index breakfast provide a beneficial impact on cognition. Such an observation indicates that cognitive improvement needs a high energy amount (Cooper et al., 2011). However, the fact that the low glycemic index breakfast is more beneficial indicates that the quality of the nutrients ingested has also a significant impact on brain activity. Thus, low glycemic index foods containing fibers or non-digestible carbohydrates, or low carbohydrates seems to have a better impact. This result supports a role for products of gut metabolism for the non-digestible carbohydrates or from other sources of energy than glucose: SCFA and ketone bodies that we will discuss in section “Putative Mechanisms at Play to Explain the Beneficial Roles of Certain Diets on Brain Function.”

Overall, it is difficult to clearly address whether diet can affect cognitive function in healthy populations after adolescence. On the other hand, there is no doubt that unhealthy dietary habits induce cognitive decline during aging and increase the risk of dementia and age-related neurological disorders. In this regard, a better knowledge of the effects of healthy diets on cognition in healthy adults becomes necessary. Such understanding would help to make better dietary recommendations toward the prevention of age-related disorders. In addition, schoolchildren studies indicate that the diet can significantly affect school performances. Hence, it should be of interest to determine whether such a role is specific to learning processes in young populations or can also influence cognitive performances in adults. It is of interest to note the results obtained on mood improvement as well. Indeed, mood disorders represent an increasing concern in modern societies and working environment (Kessler, 2006; Woo and Postolache, 2008; Hannerz et al., 2009; Firth et al., 2020). Thus, dietary improvements could be an important step to prevent the onset of depression and anxiety symptoms and contribute to better life conditions in a large part of the population.

## Healthy Diets and Brain Disorders

A certain number of brain disorders have been targeted by specific dietary interventions since decades and even centuries. For instance, the ketogenic diet (KD) is given to epileptic patients since more than 100 years (Williams and Cervenka, 2017). Furthermore, treatment for several other brain conditions now include dietary interventions (Carneiro and Leloup, 2020). Noteworthily, the knowledge of mechanisms disrupted in the disorders studied have led to several hypotheses on the diet induced mechanisms at play in the beneficial effects observed. In this section, we will describe recent research on brain disorders and the impact of food on the symptoms, progress, and outcome of the diseases. The putative mechanisms will be presented as well as the diet composition with the most significant impact.

Epilepsy is a neurological condition characterized by recurrent spontaneous seizures. These seizure episodes result from hypersynchronous discharges of neurons (Thijs et al., 2019). Notably, epileptic persons have been treated with ketogenic diets for more than 100 years with significant decreases in seizure events (Williams and Cervenka, 2017). This beneficial effect is of particular interest in drug resistant patients. Indeed, ketogenic diet intervention is associated with up to a 60% decrease of seizures in drug resistant individuals (Levy et al., 2012; Sharma and Jain, 2014a; Martin et al., 2016). Ketogenic diets have been initially used as a replacement for a fast (Wheless, 2004). Indeed, the classical ketogenic diet is a very high fat content diet (70% and above of energy from fats) and low protein and carbohydrate (10% or less). Therefore, the limited availability of sugars as energy source forces the body to use fat as the primary source of energy as it would happen during a fasting period. In the liver, this metabolic shift leads to the formation of ketone bodies from β-oxidation. However, this diet also produces long term side effects including ketoacidosis, liver lipid accumulation in long term diet, weight loss, diminished growth, kidney stones, increased blood cholesterol and fatty acid levels, while it also bears disadvantages such as a poor taste and thus aversive effects, among others. To counter those effects, several modified ketogenic diets have now been developed. Although these diets still induce ketogenesis, most of the negative effects can be dampened (Sharma and Jain, 2014b). For instance, a modified Atkins diet (the other name for the ketogenic diet), a low glycemic index diet, and medium chain triglycerides diets have also been tested with good results on epileptic patients. Although less restrictive on carbohydrates, all these modified ketogenic diets still lead to increased circulating ketone bodies levels (Verrotti et al., 2020). Mostly, these modified ketogenic diets include higher amounts of carbohydrates although still at very low levels compared to a normal diet (up to 20% instead of 55%). In addition, protein levels are less restricted and medium chain triglycerides are preferred. Overall, the amount of sugars as well as their type (complex sugars are not absorbed, but metabolized by the microbiota, therefore making glucose availability limited for host cells) appear to be key in the effect of the ketogenic diet to obtain a positive outcome on epileptic populations. These ketogenic diets have provided significant results on epileptic individuals in multiple clinical studies. Notably, the results presented show beneficial effects of the diets in a wide range of populations. Indeed, this is the case for epileptic children, adults, but also patients with GLUT1 deficiency syndrome or pyruvate dehydrogenase deficiency (both diseases are associated with seizures) (D’Andrea Meira et al., 2019). Finally, it is worth to note that if ketogenic diets are used on drug resistant patients, in regard of the significantly positive effects measured, it would be of interest to expand the ketogenic diet use to all categories of epileptic patients. However, one limitation to this global use is the poor understanding of the mechanisms involved. In addition, the ketogenic diet presents numerous negative effects that need to be considered (migraines, keto-acidosis, liver steatosis, fatigue, but also poor taste).

Interestingly, many other brain disorders have been shown to improve in patients following a similar low carbohydrate availability induced by diet strategies. Such observations support the idea of a brain metabolic shift that would compensate for decreased glucose supply.

First, high fat diet has been previously described as increasing the risk of brain stroke (Haley et al., 2019). In accordance, adjustments of the diet composition would prevent the occurrence of stroke. Thus, some studies have attempted to determine the impact of a healthy diet on stroke prevention but also during recovery. Results of such investigations clearly support a beneficial effect of adjusted diet on stroke events. Interestingly, as observed for epilepsy, low carbohydrate, ketogenic, or low glycemic index diets led to the most significant benefits (Ayuso et al., 2017). Here though, a high fructose diet is also described as a potent enhancer of stroke risk. This observation suggests that blood glucose level alone is highly important in stroke prevention. Therefore, diets with a lowering effect on glycemia or lipidemia would be beneficial for stroke prevention. In addition, the importance of a low level of blood glucose is also supported by the impact of low carbohydrate, or low glycemic index diets since they are expected to only slightly alter blood glucose levels. Accordingly, the Mediterranean diet is shown to promote stroke prevention, as well as reduce severity (Psaltopoulou et al., 2013; Lakkur and Judd, 2015; Tuttolomondo et al., 2015). Further, the vegetarian diet, a low glycemic index diet, is also associated with a decreased risk of stroke (Chiu et al., 2020). Finally, the ketogenic diet that limits the use of sugars as energy source, is described as participating to the improvement of the outcome following a stroke (Shaafi et al., 2014, 2019).

Overall, since high blood glucose is linked to an earlier stroke onset and worst outcome, a diet intervention targeting glycemia would be of interest in stroke management. Further, dietary habits that limit blood glucose increase would also help prevent stroke accidents. Hence, more research on the role of diets in stroke outcome and prevention is required. Indeed, currently, only descriptive studies have been performed. Notably, no mechanistic studies have really permitted to identify the cellular pathways involved in the positive role of healthy diets. Furthermore, in addition to diet intervention, the description of the mechanisms could help identify better pharmacological targets to improve stroke recovery.

When considering age-related disorders in general, Alzheimer’s disease (AD) is the most common cause of dementia (García-Casares et al., 2021). More recently, AD has been linked to metabolic disorders. Notably, individuals with type 2 diabetes or obesity present a higher risk of AD (Sims-Robinson et al., 2010). In addition, AD patients are characterized by a glucose uptake defect in brain cells. Interestingly, a lower risk of AD development is associated with the Mediterranean diet (Scarmeas et al., 2007). In another study, Gu et al. (2010) assessed the association of different nutrients with the prevention of AD. The authors described the putative role of ω3 and 6 polyunsaturated fatty acids (PUFA), Vitamin E and folates in the protective role of the Mediterranean diet (Gu et al., 2010). Interestingly, here, the lipid profile seems to play a key role. However, it is worth to note that the low glycemic index of such a diet could still be involved in the protective effect. Nevertheless, these results strongly support a role of brain energetics. Indeed, ω3 and 6 are well known for their role in brain activity (Yehuda, 2003). Thus, here PUFA could act as alternative energy sources for the brain in a diet limiting glucose availability. Similarly, Bayer-Carter et al. (2011), demonstrated a strong relationship between diet composition and both risk and progress of AD. As observed before, the low saturated fat diet is beneficial here as well. In addition, the authors also showed that the low fat associated to a low glycemic index diet displays the best results on cognition and AD markers measured (Bayer-Carter et al., 2011).

Similar observations can be found for Parkinson’s disease (PD) (Maraki et al., 2019). Furthermore, diet-induced hyperketonemia was described as providing benefits to PD patients (VanItallie et al., 2005). Such results confirm a key role of low glucose for brain disorders improvement. Interestingly, the Mediterranean diet is also known to decrease the risk of PD (Jackson et al., 2019). It is interesting to note the beneficial effect of the Mediterranean diet on such a wide range of brain disorders. Overall, it suggests that common mechanisms are most likely involved in the development of cognitive deficits, and putatively in aging, affecting important metabolic processes for general brain function. In support of this hypothesis, mood disorders also can be addressed through a nutritional approach showing here as well a tight relationship with metabolic control (Pervanidou et al., 2013; Hajebrahimi et al., 2016). Thus, high glycemic index diets are correlated with an increased risk of depressive disorder as well as to an increased severity of the disease (Salari-Moghaddam et al., 2019). Autism Spectrum disorder (ASD) is another neurological disorder with both behavioral and metabolic dysregulations since obesity and type 2 diabetes during pregnancy represent risk factors for this disease (Lyall et al., 2011; Krakowiak et al., 2012). Furthermore, ASD risk is expected to increase with a high glycemic index diet while a low glycemic index should decrease this risk (Currais et al., 2016; Carneiro and Leloup, 2020). After birth, dietary intervention in ASD affected newborns have shown good results. For instance, in rodent models, a ketogenic diet improves social interactions and dampens repetitive behaviors (Ruskin et al., 2013). Noteworthily, low glycemic index diets also improved ASD behaviors. Such an effect involved a change in gut microbiota which is known to be associated with ASD (van de Sande et al., 2014; Berding and Donovan, 2020; Kandeel et al., 2020).

The benefits of a low glycemic index ketogenic diet on brain function are not limited to neurodegeneration, stroke or ASD that are associated with cell death and/or cell dysfunctions. Indeed, mood disorders can also be improved by dietary interventions (Arab et al., 2019). In this situation, carbohydrates levels appear as key in the effect of the diet (Firth et al., 2020). Therefore, a long-term low carbohydrate diet exhibits positive effects on mood and cognition (Brinkworth et al., 2009).

Overall, it is interesting to note that many brain disorder conditions can be targeted by imposing a particular diet to improve the symptoms, help recovery, or prevent the onset of the disease. In addition, it appears clear that the impact of a diet on glycemia will have strong effects on brain function. However, such a result is not surprising since the brain represents at least 20% of the total body glucose consumption at rest. Hence, it is interesting to note that decreasing the availability of glucose has a positive impact on brain function in pathological conditions. Knowledge of the mechanisms at play would provide a better understanding of the diet effect but also of the disease itself. Furthermore, it could also provide a better understanding of the relationship between brain function and metabolic regulations and disorders. Furthermore, it is interesting to note that gut-brain communication seems to be central to the interaction brain function-metabolism. Indeed, several studies report the involvement of gut products in brain function and disease.

Among the different types of diet, it is interesting to note that low glycemic index (GI) diets are described as the most beneficial on health (Carneiro and Leloup, 2020). Such diets are enriched in foods with low GI, meaning that the effect on blood glucose is limited. Noteworthily, Mediterranean diet, ketogenic diet and modified ketogenic diets are all considered as low GI. Furthermore, among the diets described in the literature, these diets are the most frequently used. In fact, those diets present similar metabolic effects including gut microbiota changes favoring short Chain Fatty Acids (SCFA) production, and liver ketogenesis. Altogether, the mechanisms involved are linked to the action of these molecules in the body that will affect brain function.

## Putative Mechanisms at Play to Explain the Beneficial Roles of Certain Diets on Brain Function

Studies made in the context of epilepsy have led to several hypotheses about the mechanisms involved in the effect of the ketogenic diet to reduce seizures. Some suggested mechanisms directly involve the end product of lipid oxidation, whose synthesis is stimulated by the decrease in carbohydrate availability: ketone bodies (Guzmiirp and Geefen, 1993; Gano et al., 2014). In addition, mitochondria and gene regulation are also expected to be involved.

### Inflammation

Inflammation is a common marker found in many brain disorders including epilepsy, stroke, AD and PD. ASD is also considered at higher risk for the fetus in women suffering from inflammatory diseases during pregnancy (Theoharides et al., 2016; Raj et al., 2017; Siew et al., 2019). Interestingly, ketone bodies that are produced under most diets used in clinical studies have been demonstrated to present anti-inflammatory properties. This direct effect has been demonstrated in ischemia-induced seizures. Here, the activation by ketone bodies of the GPR109 receptor located on infiltrated macrophages in brain, stimulates the production of prostaglandins. In turn, this induces an anti-inflammatory response that protects against seizures (Rahman et al., 2014; Vezzani et al., 2016). Further, Youm et al. (2015) also observed a reduction in ischemic seizures in mice fed a ketogenic diet or receiving a brain infusion of ketone bodies. In addition, they also demonstrated that ketone bodies inhibit the assembly of NLRP3 inflammasomes by blocking the K^+^ efflux (Youm et al., 2015).

Alzheimer’s disease is also linked with inflammation (Hascup et al., 2019). Indeed, the accumulation of Aβ stimulates the recruitment of microglia and astrocytes. In parallel, interferon gamma (IFNγ), interleukin 1β (IL1β), and tumor necrosis factor α (TNF α) are secreted leading to a local inflammation (Sastre et al., 2006; Tan et al., 2013). Since microglial cells are from the same lineage than macrophages, therefore it is possible that a direct mechanism as described for stroke could also occur in AD. In fact, high caloric diets known to induce inflammation are also described to exacerbate AD. In addition, since healthy diets can decrease the Aβ accumulation, it is expected that inflammation will also be lower by dampening the recruitment of immune cells (Samadi et al., 2019). However, it is worth to note that in this case, the effect seems indirect. Thus, as suggested by Samadi et al. (2019), the effect of a normal diet compared to a healthy diet (such as Mediterranean diet) should be studied to clearly address the issue of the impact of a healthy diet independently of the high caloric negative effect already described. Autism is a neurological disorder whose risk for the offspring is increased by obesity or diabetes during pregnancy (Lyall et al., 2011; Krakowiak et al., 2012). In fact, the chronic inflammation induced by these metabolic disorders plays an important role in the increased risk of autism (Vargas et al., 2005; Patterson, 2009; Michel et al., 2012). Interestingly, this inflammatory grade can be decreased by a low glycemic index diet while a high glycemic index diet is shown to participate to the inflammatory processes (Fleming et al., 2011; Neuhouser et al., 2012; Uchiki et al., 2012). Again, in mouse models, a ketogenic diet was able to improve social behaviors (Ruskin et al., 2013). In addition, gluten-free food was shown to decrease the inflammatory grade and improved autism symptoms (Karhu et al., 2020; Sumathi et al., 2020).

**FIGURE 1:**
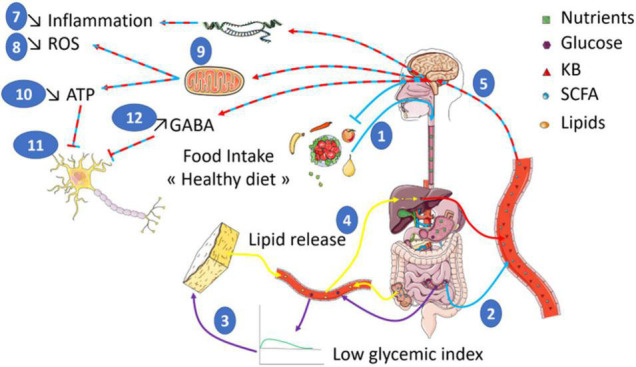
Food intake of a diet rich in low glycemic index food is shown to have a positive impact on health (1). The composition of such a diet enriched in non-digestible fibers leads to the production of SCFA (Short Chain Fatty Acids) by gut bacteria (2). In addition, the low impact on glycemia (3) will trigger an increase in KB (Ketone Bodies) production through the induced lipid oxidation increase (4). In turn, both ketone bodies and SCFA are expected to act on brain function (5) by stimulating the transcription of genes (6) involved in the reduction of inflammation (7) and ROS production (8). SCFA and KB also modulate mitochondrial activity (9) to reduce ROS production. In addition, the reduced mitochondrial respiration will diminish ATP levels (10) that would inhibit neuronal activity (11). Finally, other studies indicate increased GABA levels also contribute to the inhibition of neuronal activity (12). Altogether, such a global effect on brain bioenergetics could participate to brain benefits of healthy diet in the context of brain disorders whose neuronal function is altered.

However, it is difficult to clearly identify the mechanisms regulating inflammation. Indeed, the diets used in the studies differ widely, which makes it difficult to attribute the effects to ketone bodies only. Furthermore, some studies described direct effects while others indicated an indirect action of ketone bodies. Therefore, further investigations are essential to demonstrate the interactions between inflammation and diet since it is clear that the inflammatory grade plays an important role in neurological disorders (Figure 1).

### Redox Balance Regulation

Oxidative stress is often found in brain areas affected in the different aforementioned brain disorders (Frustaci et al., 2012; Pearson-Smith and Patel, 2017; Carneiro and Leloup, 2020). Interestingly, reactive oxygen species (ROS) production can vary depending on nutrient fluxes (Leloup et al., 2011). Therefore, not surprisingly, several studies suggested that the impact of a diet on neurological disorders involves changes in the Redox balance that could participate in the positive outcome.

Ketone bodies in particular can alter the Redox balance since they can directly enter mitochondria where a large amount of ROS can be produced within cells. In mitochondria, ketone bodies stimulate mitochondrial respiration and NADH oxidation. This in turn reduces ROS production while increasing ATP synthesis (Izzo et al., 2016). These events will prevent the mitochondrial permeability transition (mPT) and thus prevent cell death (Kim et al., 2015). Since ketone bodies affect mPT, this mechanism could participate to the anti-epileptic effect of the ketogenic diet. Indeed, mouse models of epilepsy present an increase in the threshold of mPT. In addition, mPT prevention involves modulation of cyclophilin D. Finally, in these mice it is observed a decrease of long-term potentiation and of learning and memory capacities, supporting a role in cognition (Zhou et al., 2018).

Another possible regulatory mechanism of ROS production can be due to the improved mitochondrial activity induced by ketone bodies (Cooper et al., 2018). Noteworthily, mitochondrial activity is associated with neuronal function and diseases. In addition, mitochondria are a main contributor of ROS production within cells. Therefore, the ability of ketogenic diets to decrease mitochondrial ROS production when compared to a normal chow diet is of great interest. This antioxidant property appears to come from changes in the expression of genes coding for the oxidative pathway. In addition, the NAD/NADH ratio is increased by ketone bodies. This increased ratio will in turn reduce ROS production in mitochondria and participate to the improvement of epilepsy management (Pearson-Smith and Patel, 2017).

In fact, ketone bodies can directly increase O_2_ consumption at the mitochondrial respiratory chain level. This O_2_ consumption increase will reduce ROS production. Therefore, the decrease oxidative stress associated with a lower ROS level is likely to be at play in the improvement of epilepsy observed following a ketogenic diet. Indeed, oxidative stress is associated with epileptic seizures, and therefore a decrease in oxidative stress should also help decrease the frequency of seizures (Knowles et al., 2018). In addition of decreasing ROS production, it has been also shown that a ketogenic diet stimulates the expression level of catalase. Catalase is the main antioxidant enzyme and thus participate to convert ROS into non-reactive species. Catalase expression is stimulated by the transcription factor peroxisome proliferator activated receptor γ2 (PPARγ2) (Knowles et al., 2018). In fact, in this study the authors demonstrated a regulation of the PPAR gene through histone hyper-acetylation. More precisely, it is suggested that ketone bodies inhibited histone deacetylases. In turn, PPAR upregulates antioxidant genes and downregulates pro-inflammatory genes (NFKappaB, cyclooxygenase 2, and iNOS). This hypothesis is supported by the successful use of histone deacetylase inhibitors as anti-inflammatory and anti-epileptogenic molecules (Jeong et al., 2011; Shimazu et al., 2013; Damaskos et al., 2017; Simeone et al., 2017a,b).

Stroke studies have also demonstrated the increased risk as well as the negative impact on recovery caused by high glycemia and therefore diabetes (Sander and Kearney, 2009; Lee et al., 2018). Oxidative stress is also associated with diabetes (Leloup et al., 2011). Furthermore, hyperglycemia-induced oxidative stress is expected to participate to the poor outcome following stroke. Thus, a well-balanced blood glucose level is important in the treatment and prevention of stroke (Robbins and Swanson, 2014). In this regard, diets that help maintain a low level of blood glucose contribute to a better outcome in patients with acute ischemic stroke independently of diabetes (Song et al., 2018). In the same study however, the authors suggested that chronic hyperglycemia is the main cause of stroke as it was previously proposed (Kamouchi et al., 2011; Luitse et al., 2017). Although no direct evidence related to the Redox balance is provided, it is worth to note that chronic hyperglycemia is expected to induce a chronic increase in ROS production. On the other hand, a better control of glucose levels would prevent an excessive ROS increase. Therefore, ROS levels could likely participate in the observed effects.

Finally, a similar involvement of ROS level regulation can be found in AD (Prins, 2008; Achanta and Rae, 2017). Several reports suggest that ROS production could be inhibited by an increased expression level of uncoupling proteins (U) as previously shown independently of the diet (Sullivan et al., 2004; Klaus and Ost, 2020). Therefore, the expression level of UCPs needs to be determined in healthy diets fed individuals to evaluate whether it could be another player involved in the beneficial effect of such diets. Alternatively, the ketogenic diet could also participate in the reduction of ROS production by upregulating the expression of antioxidant proteins (MnSOD, Glutathione, and Nrf2) (Taylor et al., 2019). Lastly, the decrease in neuronal activity induced by a lower energy amount provided by the diet is also hypothesized to contribute to lower ROS production and thus potentially neuroprotection (Ma et al., 2007; Figure 1).

### Blood Glucose Level

Aside end products of metabolism such as ketone bodies, the blood glucose level appears to play an important role in diseases’ risk, progress, and outcome. In fact, a rapid rise in glycemia is associated with increased oxidative stress as well as endothelial dysfunction in diabetes (Ceriello et al., 2008). Even more than hyperglycemia, excessive blood glucose level variations are more deleterious. Such high variability is one of the consequences of high glycemic index foods that induce a rapid increase in glycemia but only transiently since it is quickly regulated to reestablish a normal glycemia. This rapid rise is shown as negative for stroke recovery due to endothelial dysfunction (Santos-García et al., 2009). Furthermore, high glycemic index diets are responsible for insulin resistance that will increase serum levels of fibrinogen and von Willebrand factor involved in endothelial function, which will contribute to increase the risk of stroke (Meigs et al., 2000; Raynaud et al., 2000; Schothorst et al., 2009). In fact, a high glycemic index diet induces hypercoagulability associated with increased thrombosis risk. In parallel, small vessel diseases are also increased and participate to a negative outcome after a stroke (Song et al., 2018). Interestingly, the low glycemic index Mediterranean diet is linked to a lower risk of stroke (Spence, 2019). It was also shown that chronic episodes of hyperglycemia are associated with a decrease in cognitive function recovery. In this context, diets limiting the amplitude of glycemic variations are more beneficial on both vascular and cognitive function recovery following stroke (Lim et al., 2018).

### Neuronal Activity

Neurological disorders can be defined as brain diseases affecting neuronal activity. Such disorders are often associated with a decrease of glucose utilization. Since glucose is the main energy source for brain cells, it is thus not surprising that brain function will be altered. Therefore, diets providing ketone bodies could partly compensate this decreased glucose utilization. Indeed, it was previously observed that during glucose utilization limitation, ketone bodies can be used by the brain as an alternative energy source (Mergenthaler et al., 2013). More precisely, ketone bodies reaction with oxaloacetate produces acetyl-coA that will then stimulates the Krebs cycle. In turn, the Krebs cycle activity will increase the production of α-ketoglutarate. Then α-ketoglutarate reaction consumes aspartate, whose level is decreased in cells, and produces large amounts of glutamate. This glutamate is then decarboxylated by a glutamic acid decarboxylase which results in the synthesis of GABA (γ-aminobutyric acid), the inhibitory neurotransmitter found in the brain (Hartman et al., 2007). This metabolic loop might be very interesting in the context of stroke or epilepsy since GABA is a known anti-seizure substance targeted by some drugs that enhance GABA action (Petroff et al., 1996; Homanics et al., 1997; Olsen and Avoli, 1997; Treiman, 2001; Chuang and Reddy, 2020; Sills and Rogawski, 2020). This mechanism of action is strongly supported by the finding of high levels of GABA in the cerebrospinal fluid of children treated with low carbohydrate diets (Dahlin et al., 2005).

In addition to this indirect mechanism, ketone bodies are also expected to directly enter mitochondria and the tricarboxylic acid cycle (TCA) where they will be oxidized and thus stimulate oxidative phosphorylation. This in turn inhibits phosphofructokinase 1 and glycolysis, decreasing ATP production. A decreased ATP level will lead then to the opening of ATP-sensitive potassium channels (K_*ATP*_) and thus a decrease of neuronal activity (Ma et al., 2007). Such a hypothesis has been tested in a genetic model of drosophila presenting seizure-like activity when mechanically stimulated. This fly displays decreased seizures when given ketone bodies. Furthermore, the blockade of K_*ATP*_ channels or administration of a GABA antagonist partially reverses decreased seizure induced by ketone bodies (Li et al., 2017). However, this partial recovery suggests that other mechanisms are at play as well. Among possible mechanisms, blockade of the transfer of the vGLUT transporter (vesicular glutamate transporter) to the synapse by ketone bodies is suggested by some studies. Such a transfer inhibition would cause a decrease in glutamate excitatory activity, and thus neuronal activity inhibition (Juge et al., 2010; Omote et al., 2011). Another possible mechanism could involve the synaptic vesicle recycling. In fact, the authors showed that mice treated with ketone bodies present a reduction of both endocytosis and exocytosis combined with a misbalance between endocytosis and exocytosis. This effect is hypothesized by the authors to participate to the anticonvulsant activity of the ketogenic diet in epilepsy (Hrynevich et al., 2016; Figure 1).

### Short Chain Fatty Acids

Lastly, it is interesting to note that if most research results suggest a role for ketone bodies in the beneficial effects provided by low carbohydrate diets, other factors have more recently been suggested to play a key role as well. Therefore, microbiota has been shown to interact with brain function through the metabolism of dietary fibers in certain bacteria that produce propionate or butyrate, two SCFA (short-chain fatty acids). In fact, these SCFA exert various actions in the brain by regulating histone deacetylases, transcription factors, and antioxidant regulations. Noteworthily, all of these processes have been described to play a role in neuronal function regulation and neurodegenerative disorders. The healthy diets providing the most benefits in the context of neurological disorders are adjusted for carbohydrate contents and often enriched in dietary fibers. Therefore, it is expected that such diets could involve SCFA positive regulations while processed foods and non-healthy diets would rather have a negative impact. Indeed, many diets in industrialized countries contain high amounts of processed food, and also lead to a decrease in the number of bacteria producing SCFA. These diets are also known for their negative impact on neurodegeneration (Martínez Leo and Segura Campos, 2020). In addition, highly processed foods are likely to have a high glycemic index and therefore induce large rises in blood glucose when ingested. On the other hand, dietary fibers are low glycemic index carbohydrates which support a protective role of low glycemic index foods against neurodegenerative diseases.

In addition to neurodegenerative disorders, the gut-brain axis related to nutrition has been shown to participate in the pathophysiology of Autism, suggesting a role for the gut in general brain function and diseases (van de Sande et al., 2014; Berding and Donovan, 2020; Kandeel et al., 2020). For instance, dietary fibers induced SCFA production in the Mediterranean diet is believed to participate in the protection against Parkinson’s disease. First, SCFA can improve insulin sensitivity and reduce inflammation while stimulating BDNF (brain-derived neurotrophic factor) production. All of these factors contribute to the protection against Parkinson’s disease. The antioxidant properties of the Mediterranean diet are also expected to participate to this protection since antioxidant products can stimulate SCFA production (Jackson et al., 2019).

Nevertheless, despite several encouraging results linking gut microbiota, SCFA and brain, no clear pathway and/or mechanisms have been identified. However, it is worth to note that most of the hypotheses come back to inflammation, Redox balance or epigenetic gene regulations that have been strongly suggested as involved in the nutritional benefits to brain function and diseases. Thus, it appears worthwhile to pursue research efforts in this direction to putatively identify possible therapies or so-called “nutri-therapies.”

### Gut Microbiota

It is worth to note that diet change will affect gut microbiota composition. In recent years, many reports have highlighted the role of the microbiota on brain function and notably during brain disorders. Mediterranean diet for instance which is enriched in plant foods will likely modify the microbiome to favor fiber processing bacteria (*Akkermansia municiphilla, Ruminococcus bromii, Faecalibacterium prausnitzii, Eubacterium rectale, Eubacterium hallii, and R. bromii…)* (Morrison and Preston, 2016; Harris et al., 2021) and therefore produce SCFA. Similarly, low GI diet is a diet containing a high number of fibers. This specific composition will also favor fiber processing bacteria that will again release SCFA. Thus, the shift toward a SCFA producing microbiome would likely be beneficial in a context of neurological disorders as discussed above. Bile acids are also products affected by food composition. Briefly, high fat content increases bile acid production. These biliary acids can then be transformed by bacteria from the gut producing molecules that can interact with specific receptors found in brain to modulate satiety. Interestingly, studies have also suggested that bile acids can display antioxidant and anti-inflammatory properties. Thus, we cannot exclude a putative role of bile acids and gut microbiome in the dietary effect (Huang et al., 2022). Finally, microbiota can also produce tryptophan whose anti-inflammatory action has also been demonstrated. Overall, it is thus very likely that other factors produced by the new microbiome selected by the diet could impact brain health.

Hence, although largely studied now, the role of microbiota on brain disorders would need further attention especially in the context of diet intervention. Moreover, a specific review of the literature would be needed to extensively present the recent progress made on this topic (Yu et al., 2016; Barber et al., 2020; Beam et al., 2021; Huang et al., 2022).

## Conclusion

Altogether, it is rather likely that nutritional approaches could be significantly beneficial in a wide range of brain disorders. However, although a quite large number of studies suggests effects involving inflammation, Redox balance and mitochondrial activity, gene regulations, and neuronal activity regulation, no direct demonstration has been made so far. Furthermore, it appears that ketone bodies and SCFA produced in response to specific diets, are at the origin of the beneficial effects. However, it is important to stay cautious since ketone bodies impact on health is still not completely understood. Particularly, discrepancies exist in the literature about their positive or negative impact on metabolism. Among the putative explanations for these contradictory results, the amount of ketone bodies could play a role. In fact, a dose-dependent effect is among the hypotheses to be tested. Therefore, with the current understanding of ketone bodies effect on physiological processes, it is not possible to target the biological pathways involving ketone bodies. Nevertheless, ketogenic diets or low glycemic index diets are widely used with little side effects. Thus, focusing on the clinical outcome following such diets for individuals suffering from neurological disorders could be an interesting future research axis. Furthermore, a better understanding of the negative effects on the risks for certain disorders is also of great importance. This knowledge would be needed in order to adapt nutritional facts especially regarding sugars to improve health of aging populations in our societies (Figure 1).

**FIGURE 2:**
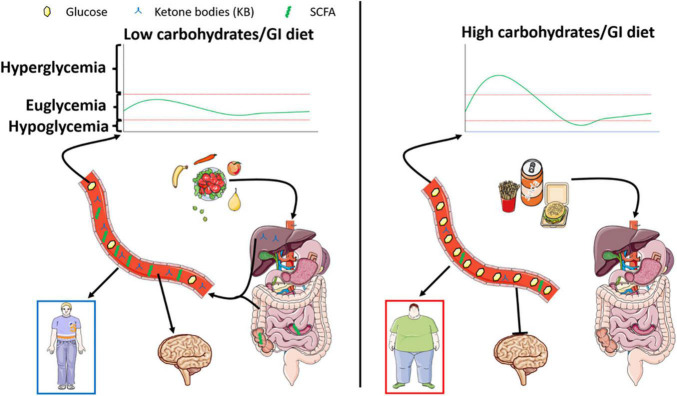
Left panel represents the effect of a low carbohydrate/glycemic index diet known for its beneficial impact on energy homeostasis. The low levels of digestible sugars are responsible for increased SCFA produced within the gut, paralleled by stimulated lipid oxidation le**a**ding to the production of ketone bodies. Both nutrients exhibited a putative beneficial role on metabolism and brain function leading to the prevention of cognitive deficits. On the other hand, represented in the right panel, a high carbohydrate/glycemic index diet will favor obesity and associated metabolic disorders. Interestingly, such diets dampen the production of ketone bodies and SCFA and are associated with decreased cognitive function.

Finally, although promising, the use of diets against diseases is also to be consider with care. Indeed, diets described in this review can also be associated with negative side effects to be taken into account. Among those negative effects, sarcopenia, weight loss, ketoacidosis, fatigue are well known. Ketogenic diets are especially known for those effects that need to be carefully controlled. In this regard, modified ketogenic diets have been recently developed to include higher amounts of carbohydrates in order to limit some of the side effects. Metabolic overload or nutritional biases are also to be mentioned when looking at these specific diets. Indeed, the high amount of fats associated with decreased carbohydrates can be either regarded as unbalanced diets. In fact, these “healthy” diets are biased since they force the organism to switch to an unnatural metabolism. Lastly, it is worth to note the aversive effect of such diets due to a low amount of carbohydrate. Indeed, taste and hedonic aspects of food intake are both part of a normal healthy feeding behavior. Therefore, use of such diets come along with increased use of non-caloric sweeteners whose effects on health are still poorly documented. Altogether, research is needed not only to completely understand the mechanisms at play in healthy diet benefits, but also to clearly determine the balance between positive and negative effects of such dietary changes in populations either healthy as preventive action, or unhealthy to provide a reduction of symptoms (Figure 2).

## Author Contributions

Both authors wrote the manuscript, contributed to the article, and approved the submitted version.

## Conflict of Interest

The authors declare that the research was conducted in the absence of any commercial or financial relationships that could be construed as a potential conflict of interest.

## Publisher’s Note

All claims expressed in this article are solely those of the authors and do not necessarily represent those of their affiliated organizations, or those of the publisher, the editors and the reviewers. Any product that may be evaluated in this article, or claim that may be made by its manufacturer, is not guaranteed or endorsed by the publisher.

## Footnotes

1
https://www.who.int/news-room/fact-sheets/detail/healthy-diet

## References

[B1] Abdelhamid A. S. Martin N. Bridges C. Brainard J. S. Wang X. Brown T. J. (2018). Polyunsaturated fatty acids for the primary and secondary prevention of cardiovascular disease. *Cochrane Database Syst. Rev.* 2018:CD012345. 10.1002/14651858.CD012345.pub2 30484282 PMC6517012

[B2] Achanta L. B. Rae C. D. (2017). β-hydroxybutyrate in the brain: one molecule, multiple mechanisms. *Neurochem. Res.* 42 35–49. 10.1007/s11064-016-2099-2 27826689

[B3] Amine E. K. Baba N. H. Belhadj M. Deurenberg-Yap M. Djazayery A. Forrestre T. (2003). Diet, nutrition and the prevention of chronic diseases. *World Health Organ. Tech. Rep. Ser*. 916 i–viii,1–149. 10.1093/ajcn/60.4.644a 12768890

[B4] Apekey T. A. Morris A. J. E. Fagbemi S. Griffiths G. J. (2009). Effects of low-fat and low-GI diets on health. *Nutr. Food Sci.* 39 663–672. 10.1108/00346650911002995

[B5] Arab A. Mehrabani S. Moradi S. Amani R. (2019). The association between diet and mood: a systematic review of current literature. *Psychiatry Res.* 271 428–437. 10.1016/j.psychres.2018.12.014 30537665

[B6] Aridi Y. S. Walker J. L. Wright O. R. L. (2017). The association between the mediterranean dietary pattern and cognitive health: a systematic review. *Nutrients* 9:674. 10.3390/nu9070674 28657600 PMC5537789

[B7] Ayuso M. I. Gonzalo-Gobernado R. Montaner J. (2017). Neuroprotective diets for stroke. *Neurochem. Int.* 107 4–10. 10.1016/j.neuint.2017.01.013 28161467

[B175] Barber T. M. Kabisch S. Pfeiffer A. F. H. Weickert M. O. (2020). The health benefits of dietary fibre. *Nutrients* 12, 1–17. 10.3390/nu12103209 33096647 PMC7589116

[B8] Bayer-Carter J. L. Green P. S. Montine T. J. Van-Fossen B. Baker L. D. Watson G. S. (2011). Diet intervention and cerebrospinal fluid biomarkers in amnestic mild cognitive impairment. *Arch. Neurol.* 68 743–752. 10.1001/archneurol.2011.125 21670398 PMC3175115

[B176] Beam A. Clinger E. Hao L. (2021). Effect of diet and dietary components on the composition of the gut microbiota. *Nutrients* 13, 1–15. 10.3390/nu13082795 34444955 PMC8398149

[B9] Berding K. Donovan S. M. (2020). Dietary patterns impact temporal dynamics of fecal microbiota composition in children with autism spectrum disorder. *Front. Nutr.* 6:193. 10.3389/fnut.2019.00193 31998741 PMC6968728

[B10] Billingsley H. E. Carbone S. Lavie C. J. (2018). Dietary fats and chronic noncommunicable diseases. *Nutrients* 10:1385. 10.3390/nu10101385 30274325 PMC6213917

[B11] Bogdanov V. B. Bogdanova O. V. Dexpert S. Delgado I. Beyer H. Aubert A. (2020). Reward-related brain activity and behavior are associated with peripheral ghrelin levels in obesity. *Psychoneuroendocrinology* 112:104520. 10.1016/j.psyneuen.2019.104520 31786481

[B12] Bremner J. D. Moazzami K. Wittbrodt M. T. Nye J. A. Lima B. B. Gillespie C. F. (2020). Diet, stress and mental health. *Nutrients* 12:2428. 10.3390/nu12082428 32823562 PMC7468813

[B13] Brinkworth G. D. Buckley J. D. Noakes M. Clifton P. M. Wilson C. J. (2009). Long-term effects of a very low-carbohydrate diet and a low-fat diet on mood and cognitive function. *Arch. Internal Med.* 169 1873–1880. 10.1001/archinternmed.2009.329 19901139

[B14] Carneiro L. Leloup C. (2020). Mens sana in corpore sano: does the glycemic index have a role to play? *Nutrients* 12:2989. 10.3390/nu12102989 33003562 PMC7599769

[B15] Castanon N. Luheshi G. Layé S. (2015). Role of neuroinflammation in the emotional and cognitive alterations displayed by animal models of obesity. *Front. Neurosci.* 9:229. 10.3389/fnins.2015.00229 26190966 PMC4490252

[B16] Ceriello A. Esposito K. Piconi L. Ihnat M. A. Thorpe J. E. Testa R. (2008). Oscillating glucose is more deleterious to endothelial function and oxidative stress than mean glucose in normal and type 2 diabetic patients. *Diabetes* 57 1349–1354. 10.2337/db08-0063 18299315

[B17] Chamari K. Briki W. Farooq A. Patrick T. Belfekih T. Herrera C. P. (2016). Impact of Ramadan intermittent fasting on cognitive function in trained cyclists: a pilot study. *Biol. Sport* 33 49–56. 10.5604/20831862.1185888 26985134 PMC4786586

[B18] Charisis S. Ntanasi E. Yannakoulia M. Anastasiou C. A. Kosmidis M. H. Dardiotis E. (2021). Mediterranean diet and risk for dementia and cognitive decline in a Mediterranean population. *J. Am. Geriatr. Soc.* 69 1548–1559. 10.1111/jgs.17072 33724444

[B19] Chiu T. H. T. Chang H. R. Wang L. Y. Chang C. C. Lin M. N. Lin C. L. (2020). Vegetarian diet and incidence of total, ischemic, and hemorrhagic stroke in 2 cohorts in Taiwan. *Neurology* 94 1112–1121. 10.1212/WNL.0000000000009093 32102976 PMC7220235

[B20] Chuang S. H. Reddy D. S. (2020). Isobolographic analysis of antiseizure activity of the GABA Type A receptor-modulating synthetic neurosteroids brexanolone and ganaxolone with tiagabine and midazolam. *J. Pharmacol. Exp. Ther.* 372 285–298. 10.1124/jpet.119.261735 31843812 PMC7011113

[B21] Cooper M. A. McCoin C. Pei D. Thyfault J. P. Koestler D. Wright D. E. (2018). Reduced mitochondrial reactive oxygen species production in peripheral nerves of mice fed a ketogenic diet. *Exp. Physiol.* 103 1206–1212. 10.1113/EP087083 30088302 PMC6119112

[B22] Cooper S. B. Bandelow S. Nevill M. E. (2011). Breakfast consumption and cognitive function in adolescent schoolchildren. *Physiol. Behav.* 103 431–439. 10.1016/j.physbeh.2011.03.018 21439306

[B23] Cooper S. B. Bandelow S. Nute M. L. Morris J. G. Nevill M. E. (2012). Breakfast glycaemic index and cognitive function in adolescent school children. *Br. J. Nutr.* 107 1823–1832. 10.1017/S0007114511005022 22017815

[B24] Cooper S. B. Bandelow S. Nute M. L. Morris J. G. Nevill M. E. (2015). Breakfast glycaemic index and exercise: combined effects on adolescents’ cognition. *Physiol. Behav.* 139 104–111. 10.1016/j.physbeh.2014.11.024 25446221

[B25] Cordner Z. A. Tamashiro K. L. K. (2015). Effects of high-fat diet exposure on learning & memory. *Physiol. Behav.* 152 363–371. 10.1016/j.physbeh.2015.06.008 26066731 PMC5729745

[B26] Currais A. Farrokhi C. Dargusch R. Goujon-Svrzic M. Maher P. (2016). Dietary glycemic index modulates the behavioral and biochemical abnormalities associated with autism spectrum disorder. *Mol. Psychiatry* 21 426–436. 10.1038/mp.2015.64 26055422

[B27] D’Alessandro A. de Pergola G. Silvestris F. (2016). Mediterranean diet and cancer risk: an open issue. *Int. J. Food Sci. Nutr.* 67 593–605. 10.1080/09637486.2016.1191444 27251477

[B28] D’Andrea Meira I. Romão T. T. do Prado H. J. P. Krüger L. T. Pires M. E. P. da Conceição P. O. (2019). Ketogenic diet and epilepsy: what we know so far. *Front. Neurosci.* 13:5. 10.3389/fnins.2019.00005 30760973 PMC6361831

[B29] Dahlin M. Elfving Å Ungerstedt U. Åmark P. (2005). The ketogenic diet influences the levels of excitatory and inhibitory amino acids in the CSF in children with refractory epilepsy. *Epilepsy Res.* 64 115–125. 10.1016/j.eplepsyres.2005.03.008 15961283

[B30] Damaskos C. Valsami S. Kontos M. Spartalis E. Kalampokas T. Kalampokas E. (2017). Histone deacetylase inhibitors: an attractive therapeutic strategy against breast cancer. *Anticancer Res.* 37 35–46. 10.21873/anticanres.11286 28011471

[B31] Davis J. A. Paul J. R. McMeekin L. J. Nason S. R. Antipenko J. P. Yates S. D. (2020). High-fat and high-sucrose diets impair time-of-day differences in spatial working memory of male mice. *Obesity* 28 2347–2356. 10.1002/oby.22983 33043637 PMC7686286

[B32] Dohrmann D. D. Putnik P. Bursać Kovačević D. Simal-Gandara J. Lorenzo J. M. Barba F. J. (2019). Japanese, mediterranean and argentinean diets and their potential roles in neurodegenerative diseases. *Food Res. Int.* 120 464–477. 10.1016/j.foodres.2018.10.090 31000263

[B33] Dolu N. Yüksek A. Sizer A. Alay M. (2007). arousal and continuous attention during ramadan intermittent fasting. *J. Basic Clin. Physiol. Pharmacol.* 18 315–322. 10.1515/JBCPP.2007.18.4.315 18380173

[B34] Duan Y. Zeng L. Zheng C. Song B. Li F. Kong X. (2018). Inflammatory links between high fat diets and diseases. *Front. Immunol.* 9:2649. 10.3389/fimmu.2018.02649 30483273 PMC6243058

[B35] Edefonti V. Rosato V. Parpinel M. Nebbia G. Fiorica L. Fossali E. (2014). The effect of breakfast composition and energy contribution on cognitive and academic performance: a systematic review. *Am. J. Clin. Nutr.* 100 626–656. 10.3945/ajcn.114.083683 24808492

[B36] Finkel T. Serrano M. Blasco M. A. (2007). The common biology of cancer and ageing. *Nature* 448 767–774. 10.1038/nature05985 17700693

[B37] Firth J. Firth J. Gangwisch J. E. Gangwisch J. E. Borisini A. Wootton R. E. (2020). Food and mood: how do diet and nutrition affect mental wellbeing? *BMJ* 369:m2382. 10.1136/bmj.m2382 32601102 PMC7322666

[B38] Fleming T. H. Humpert P. M. Nawroth P. P. Bierhaus A. (2011). Reactive metabolites and AGE/RAGE-mediated cellular dysfunction affect the aging process – A mini-review. *Gerontology* 57 435–443. 10.1159/000322087 20962515

[B39] Frustaci A. Neri M. Cesario A. Adams J. B. Domenici E. Dalla Bernardina B. (2012). Oxidative stress-related biomarkers in autism: systematic review and meta-analyses. *Free Rad. Biol. Med.* 52 2128–2141. 10.1016/j.freeradbiomed.2012.03.011 22542447

[B40] Gano L. B. Patel M. Rho J. M. (2014). Ketogenic diets, mitochondria, and neurological diseases. *J. Lipid Res.* 55 2211–2228. 10.1194/jlr.R048975 24847102 PMC4617125

[B41] García-Casares N. Gallego Fuentes P. Barbancho M. Á López-Gigosos R. García-Rodríguez A. Gutiérrez-Bedmar M. (2021). Alzheimer’s disease, mild cognitive impairment and mediterranean diet. A systematic review and dose-response meta-analysis. *J. Clin. Med.* 10:4642. 10.3390/jcm10204642 34682764 PMC8537524

[B42] Gómez-Pinilla F. (2008). Brain foods: the effects of nutrients on brain function. *Nat. Rev. Neurosci.* 9 568–578. 10.1038/nrn2421 18568016 PMC2805706

[B43] Gomez-Pinilla F. Cipolat R. P. Royes L. F. F. (2021). Dietary fructose as a model to explore the influence of peripheral metabolism on brain function and plasticity. *Biochim. Biophys. Acta Mol. Basis Dis.* 1867:166036. 10.1016/j.bbadis.2020.166036 33508421

[B44] Gu Y. Nieves J. W. Stern Y. Luchsinger J. A. Scarmeas N. (2010). Food combination and alzheimer disease risk: a protective diet. *Arch. Neurol.* 67 699–706. 10.1001/archneurol.2010.84 20385883 PMC3029147

[B45] Guzmiirp M. Geefen M. J. (1993). Regulation of fatty acid oxidation in mammalian liver. *Biochim. Biophys. Acta* 1167 227–241. 8097629 10.1016/0005-2760(93)90224-w

[B46] Hajebrahimi B. Kiamanesh A. Asgharnejad Farid A. A. Asadikaram G. (2016). Type 2 diabetes and mental disorders; A plausible link with inflammation. *Cell. Mol. Biol.* 62 71–77. 10.14715/cmb/2016.62.13.13 28040067

[B47] Haley M. J. Krishnan S. Burrows D. de Hoog L. Thakrar J. Schiessl I. (2019). Acute high-fat feeding leads to disruptions in glucose homeostasis and worsens stroke outcome. *J. Cereb. Blood Flow Metab.* 39 1026–1037. 10.1177/0271678X17744718 29171775 PMC6545621

[B48] Hannerz H. Tüchsen F. Pedersen B. H. Dyreborg J. Rugulies R. Albertsen K. (2009). Work-relatedness of mood disorders in Denmark. *Scand. J. Work Environ. Health* 35 294–300. 10.5271/sjweh.1329 19436922

[B49] Harris H. C. Morrison D. J. Edwards C. A. (2021). Impact of the source of fermentable carbohydrate on SCFA production by human gut microbiota in vitro - a systematic scoping review and secondary analysis. *Crit. Rev. Food Sci. Nutr.* 61 3892–3903. 10.1080/10408398.2020.1809991 32865002

[B50] Hartman A. L. Gasior M. Vining E. P. G. Rogawski M. A. (2007). The neuropharmacology of the ketogenic diet. *Pediatr. Neurol.* 36 281–292. 10.1016/j.pediatrneurol.2007.02.008 17509459 PMC1940242

[B51] Hascup E. R. Broderick S. O. Russell M. K. Fang Y. Bartke A. Boger H. A. (2019). Diet-induced insulin resistance elevates hippocampal glutamate as well as VGLUT1 and GFAP expression in AβPP/PS1 Mice HHS Public Access. *J. Neurochem.* 148 219–237. 10.13140/RG.2.2.11180.10888 30472734 PMC6438176

[B52] Homanics G. E. Delorey T. M. Firestone L. L. Quinlan J. J. Handforth A. Harrison N. L. (1997). Mice devoid of-aminobutyrate type A receptor 3 subunit have epilepsy, cleft palate, and hypersensitive behavior (gene targetingbenzodiazepineAngelman syndromeanesthesia). *Neurobiology* 94 4143–4148. 10.1073/pnas.94.8.4143 PMC20582 9108119

[B53] Hrynevich S. V. Waseem T. V. Hébert A. Pellerin L. Fedorovich S. V. (2016). β-Hydroxybutyrate supports synaptic vesicle cycling but reduces endocytosis and exocytosis in rat brain synaptosomes. *Neurochem. Int.* 93 73–81. 10.1016/j.neuint.2015.12.014 26748385

[B54] Huang F. Pariante C. M. Borsini A. (2022). From dried bear bile to molecular investigation: a systematic review of the effect of bile acids on cell apoptosis, oxidative stress and inflammation in the brain, across pre-clinical models of neurological, neurodegenerative and neuropsychiatric disorders. *Brain Behav. Immun.* 99 132–146. 10.1016/j.bbi.2021.09.021 34601012

[B55] Izzo V. Bravo-San Pedro J. M. Sica V. Kroemer G. Galluzzi L. (2016). Mitochondrial permeability transition: new findings and persisting uncertainties. *Trends Cell Biol.* 26 655–667. 10.1016/j.tcb.2016.04.006 27161573

[B56] Jackson A. Forsyth C. B. Shaikh M. Voigt R. M. Engen P. A. Ramirez V. (2019). Diet in Parkinson’s disease: critical role for the microbiome. *Front. Neurol.* 10:1245. 10.3389/fneur.2019.01245 31920905 PMC6915094

[B57] Jeong E. A. Jeon B. T. Shin H. J. Kim N. Lee D. H. Kim H. J. (2011). Ketogenic diet-induced peroxisome proliferator-activated receptor-γ activation decreases neuroinflammation in the mouse hippocampus after kainic acid-induced seizures. *Exp. Neurol.* 232 195–202. 10.1016/j.expneurol.2011.09.001 21939657

[B58] Juge N. Gray J. A. Omote H. Miyaji T. Inoue T. Hara C. (2010). Metabolic control of vesicular glutamate transport and release. *Neuron* 68 99–112. 10.1016/j.neuron.2010.09.002 20920794 PMC2978156

[B59] Kamouchi M. Matsuki T. Hata J. Kuwashiro T. Ago T. Sambongi Y. (2011). Prestroke glycemic control is associated with the functional outcome in acute ischemic stroke: the fukuoka stroke registry. *Stroke* 42 2788–2794. 10.1161/STROKEAHA.111.617415 21817134

[B60] Kandeel W. A. Meguid N. A. Bjørklund G. Eid E. M. Farid M. Mohamed S. K. (2020). Impact of clostridium bacteria in children with autism spectrum disorder and their anthropometric measurements. *J. Mol. Neurosci.* 70 897–907. 10.1007/s12031-020-01482-2 32130666

[B61] Kanoski S. E. Davidson T. L. (2011). Western diet consumption and cognitive impairment: links to hippocampal dysfunction and obesity. *Physiol. Behav.* 103 59–68. 10.1016/j.physbeh.2010.12.003 21167850 PMC3056912

[B62] Karhu E. Zukerman R. Eshraghi R. S. Mittal J. Deth R. C. Castejon A. M. (2020). Nutritional interventions for autism spectrum disorder. *Nutr. Rev.* 78 515–531. 10.1093/nutrit/nuz092 31876938

[B63] Kessler R. (2006). Prevalence and effects of mood disorders on work performance in a nationally representative sample of U.S. Workers. *Am. J. Psychiatry* 163 1561–1568. 10.1176/appi.ajp.163.9.1561 16946181 PMC1924724

[B64] Kim D. Y. Simeone K. A. Simeone T. A. Pandya J. D. Wilke J. C. Ahn Y. (2015). Ketone bodies mediate antiseizure effects through mitochondrial permeability transition. *Ann. Neurol.* 78 77–87. 10.1002/ana.24424 25899847 PMC4480159

[B65] Klaus S. Ost M. (2020). Mitochondrial uncoupling and longevity – A role for mitokines? *Exp. Gerontol.* 130:110796. 10.1016/j.exger.2019.110796 31786315

[B66] Klement R. J. Pazienza V. (2019). Impact of different types of diet on gut microbiota profiles and cancer prevention and treatment. *Medicina (Lithuania)* 55:84. 10.3390/medicina5504008 PMC6524347 30934960

[B67] Knowles S. Budney S. Deodhar M. Matthews S. A. Simeone K. A. Simeone T. A. (2018). Ketogenic diet regulates the antioxidant catalase via the transcription factor PPARγ2. *Epilepsy Res.* 147 71–74. 10.1016/j.eplepsyres.2018.09.009 30261354 PMC6192850

[B68] Krakowiak P. Walker C. K. Bremer A. A. Baker A. S. Ozonoff S. Hansen R. L. (2012). Maternal metabolic conditions and risk for autism and other neurodevelopmental disorders. *Pediatrics* 129 1121–1128. 10.1542/peds.2011-2583 22492772 PMC3340592

[B69] Lakkur S. Judd S. E. (2015). Diet and stroke: recent evidence supporting a mediterranean-style diet and food in the primary prevention of stroke. *Stroke* 46 2007–2011. 10.1161/STROKEAHA.114.006306 25967574 PMC4479964

[B70] Lee K. J. Lee J. S. Jung K. H. (2018). Interactive effect of acute and chronic glycemic indexes for severity in acute ischemic stroke patients. *BMC Neurol.* 18:105. 10.1186/s12883-018-1109-1 30075761 PMC6091005

[B71] Leloup C. Casteilla L. Carrière A. Galinier A. Benani A. Carneiro L. (2011). Balancing mitochondrial redox signaling: a key point in metabolic regulation. *Antioxid. Redox Signal.* 14 519–530. 10.1089/ars.2010.3424 20977349

[B72] Levy R. G. Cooper P. N. Giri P. Weston J. (2012). Ketogenic diet and other dietary treatments for epilepsy. *Cochrane Database Syst. Rev.* 2012 1–54. 10.1002/14651858.CD001903.pub2 22419282

[B73] Li J. O’Leary E. I. Tanner G. R. (2017). The ketogenic diet metabolite beta-hydroxybutyrate (β-HB) reduces incidence of seizure-like activity (SLA) in a K atp- and GABA b-dependent manner in a whole-animal Drosophila melanogaster model. *Epilepsy Res.* 133 6–9. 10.1016/j.eplepsyres.2017.04.003 28395176

[B74] Li R. J. Liu Y. Liu H. Q. Li J. (2020). Ketogenic diets and protective mechanisms in epilepsy, metabolic disorders, cancer, neuronal loss, and muscle and nerve degeneration. *J. Food Biochem.* 44:e13140. 10.1111/jfbc.13140 31943235

[B75] Lim J. S. Kim C. Oh M. S. Lee J. H. Jung S. Jang M. U. (2018). Effects of glycemic variability and hyperglycemia in acute ischemic stroke on post-stroke cognitive impairments. *J. Diabetes Complications* 32 682–687. 10.1016/j.jdiacomp.2018.02.006 29793824

[B76] Luengo A. Gui D. Y. vander Heiden M. G. (2017). Targeting metabolism for cancer therapy. *Cell Chem. Biol.* 24 1161–1180. 10.1016/j.chembiol.2017.08.028 28938091 PMC5744685

[B77] Luitse M. J. A. Velthuis B. K. Kappelle L. J. van der Graaf Y. Biessels G. J. (2017). Chronic hyperglycemia is related to poor functional outcome after acute ischemic stroke. *Int. J. Stroke* 12 180–186. 10.1177/1747493016676619 27784821

[B78] Lyall K. Pauls D. L. Santangelo S. Spiegelman D. Ascherio A. (2011). Maternal early life factors associated with hormone levels and the risk of having a child with an autism spectrum disorder in the nurses health study II. *J. Autism Dev. Disord.* 41 618–627. 10.1007/s10803-010-1079-7 20700638 PMC3494408

[B79] Ma W. Berg J. Yellen G. (2007). Ketogenic diet metabolites reduce firing in central neurons by opening KATP channels. *J. Neurosci.* 27 3618–3625. 10.1523/JNEUROSCI.0132-07.2007 17409226 PMC6672398

[B80] Maraki M. I. Yannakoulia M. Stamelou M. Stefanis L. Xiromerisiou G. Kosmidis M. H. (2019). Mediterranean diet adherence is related to reduced probability of prodromal Parkinson’s disease. *Mov. Disord.* 34 48–57. 10.1002/mds.27489 30306634

[B81] Martin K. Jackson C. F. Levy R. G. Cooper P. N. (2016). Ketogenic diet and other dietary treatments for epilepsy. *Cochrane Database Syst. Rev.* 2016 1–54. 10.1002/14651858.CD001903.pub3 26859528

[B82] Martínez Leo E. E. Segura Campos M. R. (2020). Effect of ultra-processed diet on gut microbiota and thus its role in neurodegenerative diseases. *Nutrition* 71:110609. 10.1016/j.nut.2019.110609 31837645

[B83] Martinez-Gonzalez M. A. Martin-Calvo N. (2016). Mediterranean diet and life expectancy; Beyond olive oil, fruits, and vegetables. *Curr. Opin. Clin. Nutr. Metab0 Care* 19 401–407. 10.1097/MCO.0000000000000316 27552476 PMC5902736

[B84] Mattson M. P. (2019). An evolutionary perspective on why food overconsumption impairs cognition. *Trends Cogn. Sci.* 23 200–212. 10.1016/j.tics.2019.01.003 30670325 PMC6412136

[B85] Mattson M. P. Longo V. D. Harvie M. (2017). Impact of intermittent fasting on health and disease processes. *Ageing Res. Rev.* 39 46–58. 10.1016/j.arr.2016.10.005 27810402 PMC5411330

[B86] Mattson M. P. Moehl K. Ghena N. Schmaedick M. Cheng A. (2018). Intermittent metabolic switching, neuroplasticity and brain health. *Nat. Rev. Neurosci.* 19 81–94. 10.1038/nrn.2017.156 29321682 PMC5913738

[B87] McMillan L. Owen L. Kras M. Scholey A. (2011). Behavioural effects of a 10-day Mediterranean diet. Results from a pilot study evaluating mood and cognitive performance. *Appetite* 56 143–147. 10.1016/j.appet.2010.11.149 21115083

[B88] Meigs J. B. Mittleman M. A. Nathan D. M. Tofler G. H. Singer D. E. Murphy-Sheehy P. M. (2000). Hyperinsulinemia, hyperglycemia, and impaired hemostasis the framingham offspring study. *JAMA* 283 221–228. 10634338 10.1001/jama.283.2.221

[B89] Meldrum D. R. Morris M. A. Gambone J. C. (2017). Obesity pandemic: causes, consequences, and solutions—but do we have the will? *Fertil. Steril.* 107 833–839. 10.1016/j.fertnstert.2017.02.104 28292617

[B90] Meo S. A. Hassan A. (2015). Physiological changes during fasting in Ramadan. *J. Pakistan Med. Assoc.* 65 S6–S13. 26013791

[B91] Mergenthaler P. Lindauer U. Dienel G. A. Meisel A. (2013). Sugar for the brain: the role of glucose in physiological and pathological brain function. *Trends Neurosci.* 36 587–597. 10.1016/j.tins.2013.07.001 23968694 PMC3900881

[B92] Micha R. Rogers P. J. Nelson M. (2010). The glycaemic potency of breakfast and cognitive function in school children. *Eur. J. Clin. Nutr.* 64 948–957. 10.1038/ejcn.2010.96 20571500

[B93] Micha R. Rogers P. J. Nelson M. (2011). Glycaemic index and glycaemic load of breakfast predict cognitive function and mood in school children: a randomised controlled trial. *Br. J. Nutr.* 106 1552–1561. 10.1017/S0007114511002303 21736777

[B94] Michel M. Schmidt M. J. Mirnics K. (2012). Immune system gene dysregulation in autism and schizophrenia. *Dev. Neurobiol.* 72 1277–1287. 10.1002/dneu.22044 22753382 PMC3435446

[B95] Mittelman S. D. (2020). The role of diet in cancer prevention and chemotherapy efficacy. *Annu. Rev. Nutr.* 40 273–297. 10.1146/annurev-nutr-013120-041149 32543948 PMC8546934

[B96] Morrison D. J. Preston T. (2016). Formation of short chain fatty acids by the gut microbiota and their impact on human metabolism. *Gut Microbes* 7 189–200. 10.1080/19490976.2015.1134082 26963409 PMC4939913

[B97] Moynihan P. Petersen P. E. (2004). Diet, nutrition and the prevention of dental diseases. *Public Health Nutr.* 7 201–226. 10.1079/phn2003589 14972061

[B98] Murakami K. Miyake Y. Sasaki S. Tanaka K. Fukushima W. Kiyohara C. (2010). Dietary glycemic index is inversely associated with the risk of Parkinson’s disease: a case-control study in Japan. *Nutrition* 26 515–521. 10.1016/j.nut.2009.05.021 19628370

[B99] Naveed S. Lakka T. Haapala E. A. (2020). An overview on the associations between health behaviors and brain health in children and adolescents with special reference to diet quality. *Int. J. Environ. Res. Public Health* 17:953. 10.3390/ijerph17030953 32033054 PMC7037721

[B100] Neuhouser M. L. Schwarz Y. Wang C. Breymeyer K. Coronado G. Wang C. Y. (2012). A low-glycemic load diet reduces serum C-reactive protein and modestly increases adiponectin in overweight and obese adults. *J. Nutr.* 142 369–374. 10.3945/jn.111.149807 22190020 PMC3260063

[B101] Olsen R. W. Avoli M. (1997). GABA an d Epileptogenesis. *Epilepsia* 38 399–407. 9118844 10.1111/j.1528-1157.1997.tb01728.x

[B102] Omote H. Miyaji T. Juge N. Moriyama Y. (2011). Vesicular neurotransmitter transporter: bioenergetics and regulation of glutamate transport. *Biochemistry* 50 5558–5565. 10.1021/bi200567k 21612282

[B103] Paoli A. (2014). Ketogenic diet for obesity: friend or foe? *Int. J. Environ. Res. Public Health* 11 2092–2107. 10.3390/ijerph110202092 24557522 PMC3945587

[B104] Patterson P. H. (2009). Immune involvement in schizophrenia and autism: etiology, pathology and animal models. *Behav. Brain Res.* 204 313–321. 10.1016/j.bbr.2008.12.016 19136031

[B105] Pearson-Smith J. N. Patel M. (2017). Metabolic dysfunction and oxidative stress in epilepsy. *Int. J. Mol. Sci.* 18:2365. 10.3390/ijms18112365 29117123 PMC5713334

[B106] Pellerin L. Magistretti P. J. (2012). Sweet sixteen for ANLS. *J. Cereb. Blood Flow Metab.* 32 1152–1166. 10.1038/jcbfm.2011.149 22027938 PMC3390819

[B107] Pervanidou P. Bastaki D. Chouliaras G. Papanikolaou K. Laios E. Kanaka-Gantenbein C. (2013). Circadian cortisol profiles, anxiety and depressive symptomatology, and body mass index in a clinical population of obese children. *Stress* 16 34–43. 10.3109/10253890.2012.689040 22545868

[B108] Peters A. (2011). The selfish brain: competition for energy resources. *Am. J. Hum. Biol.* 23 29–34. 10.1002/ajhb.21106 21080380

[B109] Petroff O. A. C. Rothman D. L. Behar K. L. Mattson R. H. (1996). Low brain GABA level is associated with poor seizure control. *Ann. Neurol.* 40 908–911. 10.1002/ana.410400613 9007096

[B110] Poli A. Agostoni C. Graffigna G. Bosio C. Donini L. M. Marangoni F. (2019). The complex relationship between diet, quality of life and life expectancy: a narrative review of potential determinants based on data from Italy. *Eat. Weight Disord.* 24 411–419. 10.1007/s40519-018-0582-2 30264391

[B111] Prins M. L. (2008). Cerebral metabolic adaptation and ketone metabolism after brain injury. *J. Cereb. Blood Flow Metab.* 28 1–16. 10.1038/sj.jcbfm.9600543 17684514 PMC2857668

[B112] Psaltopoulou T. Sergentanis T. N. Panagiotakos D. B. Sergentanis I. N. Kosti R. Scarmeas N. (2013). Mediterranean diet, stroke, cognitive impairment, and depression: a meta-analysis. *Ann. Neurol.* 74 580–591. 10.1002/ana.23944 23720230

[B113] Rahman M. Muhammad S. Khan M. A. Chen H. Ridder D. A. Müller-Fielitz H. (2014). The b-hydroxybutyrate receptor HCA 2 activates a neuroprotective subset of macrophages. *Nat. Commun.* 5:3944. 10.1038/ncomms4944 24845831

[B114] Raj D. Yin Z. Breur M. Doorduin J. Holtman I. R. Olah M. (2017). Increased white matter inflammation in aging- and alzheimer’s disease brain. *Front. Mol. Neurosci.* 10:206. 10.3389/fnmol.2017.00206 28713239 PMC5492660

[B115] Raynaud E. Pérez-Martin A. Brun J.-F. Aïssa-Benhaddad A. Fédou C. Mercier J. (2000). Relationships between fibrinogen and insulin resistance. *Atherosclerosis* 150 365–370. 10856528 10.1016/s0021-9150(99)00373-1

[B116] Reddavide R. Rotolo O. Caruso M. G. Stasi E. Notarnicola M. Miraglia C. (2018). The role of diet in the prevention and treatment of inflammatory bowel diseases. *Acta Biomed.* 89 60–75. 10.23750/abm.v89i9-S.7952 30561397 PMC6502201

[B117] Robbins N. M. Swanson R. A. (2014). Opposing effects of glucose on stroke and reperfusion injury: acidosis, oxidative stress, and energy metabolism. *Stroke* 45 1881–1886. 10.1161/STROKEAHA.114.004889 24743441 PMC4102697

[B118] Rodopaios N. E. Mougios V. Konstantinidou A. Iosifidis S. Koulouri A. A. Vasara E. (2019). Effect of periodic abstinence from dairy products for approximately half of the year on bone health in adults following the Christian Orthodox Church fasting rules for decades. *Arch. Osteopor.* 14:68. 10.1007/s11657-019-0625-y 31243579

[B119] Ruskin D. N. Svedova J. Cote J. L. Sandau U. Rho J. M. Kawamura M. (2013). Ketogenic diet improves core symptoms of autism in BTBR Mice. *PLoS One* 8:e65021. 10.1371/journal.pone.0065021 23755170 PMC3673987

[B120] Safouris A. Tsivgoulis G. Sergentanis T. Psaltopoulou T. (2015). Mediterranean diet and risk of dementia. *Curr. Alzheimer Res.* 12 736–744. 10.2174/1567205012666150710114430 26159192

[B121] Salari-Moghaddam A. Saneei P. Larijani B. Esmaillzadeh A. (2019). Glycemic index, glycemic load, and depression: a systematic review and meta-analysis. *Eur. J. Clin. Nutr.* 73 356–365. 10.1038/s41430-018-0258-z 30054563

[B122] Samadi M. Moradi S. Moradinazar M. Mostafai R. Pasdar Y. (2019). Dietary pattern in relation to the risk of Alzheimer’s disease: a systematic review. *Neurol. Sci.* 40 2031–2043. 10.1007/s10072-019-03976-3 31240575

[B123] Sander D. Kearney M. T. (2009). Reducing the risk of stroke in type 2 diabetes: pathophysiological and therapeutic perspectives. *J. Neurol.* 256 1603–1619. 10.1007/s00415-009-5143-1 19399381

[B124] Sanders T. A. B. (2014). Protective effects of dietary PUFA against chronic disease: evidence from epidemiological studies and intervention trials. *Proc. Nutr. Soc.* 73 73–79. 10.1017/S0029665113003789 24308351

[B125] Santos-García D. Blanco M. Serena J. Arias S. Millán M. Rodríguez-Yáñez M. (2009). Brachial arterial flow mediated dilation in acute ischemic stroke. *Eur. J. Neurol.* 16 684–690. 10.1111/j.1468-1331.2009.02564.x 19236459

[B126] Sastre M. Klockgether T. Heneka M. T. (2006). Contribution of inflammatory processes to Alzheimer’s disease: molecular mechanisms. *Int. J. Dev. Neurosci.* 24 167–176. 10.1016/j.ijdevneu.2005.11.014 16472958

[B127] Scarmeas N. Luchsinger J. A. Mayeux R. Stern Y. (2007). Mediterranean diet and Alzheimer disease mortality. *Neurology* 69 1084–1093. 10.1212/01.wnl.0000277320.50685.7c 17846408 PMC2673956

[B128] Schothorst E. M. Bunschoten A. Schrauwen P. Mensink R. P. Keijer J. (2009). Effects of a high-fat, low- versus high-glycemic index diet: retardation of insulin resistance involves adipose tissue modulation. *FASEB J.* 23 1092–1101. 10.1096/fj.08-117119 19029198

[B129] Shaafi S. Mahmoudi J. Pashapour A. Farhoudi M. Sadigh-Eteghad S. Akbari H. (2014). Ketogenic diet provides neuroprotective effects against ischemic stroke neuronal damages. *Adv. Pharm. Bull.* 4 479–481. 10.5681/apb.2014.071 25671178 PMC4312394

[B130] Shaafi S. Sharifi-Bonab M. Ghaemian N. Mokhtarkhani M. Akbari H. (2019). Early motor-behavioral outcome of ischemic stroke with ketogenic diet preconditioning: interventional animal study. *J. Stroke Cerebrovasc. Dis.* 28 1032–1039. 10.1016/j.jstrokecerebrovasdis.2018.12.024 30658953

[B131] Sharma S. Jain P. (2014a). The ketogenic diet and other dietary treatments for refractory epilepsy in children. *Ann. Indian Acad. Neurol.* 17 253–259. 10.4103/0972-2327.138471 25221391 PMC4162008

[B132] Sharma S. Jain P. (2014b). The modified atkins diet in refractory epilepsy. *Epilepsy Res. Treat.* 2014:404202. 10.1155/2014/404202 24627806 PMC3929267

[B133] Shimazu T. Hirschey M. D. Newman J. He W. Shirakawa K. Le Moan N. (2013). Suppression of oxidative stress by β-hydroxybutyrate, an endogenous histone deacetylase inhibitor. *Science* 339 211–214. 10.1126/science.1227166 23223453 PMC3735349

[B134] Siew J. J. Chen H. M. Chen H. Y. Chen H. L. Chen C. M. Soong B. W. (2019). Galectin-3 is required for the microglia-mediated brain inflammation in a model of Huntington’s disease. *Nat. Commun.* 10:3473. 10.1038/s41467-019-11441-0 31375685 PMC6677843

[B135] Sills G. J. Rogawski M. A. (2020). Mechanisms of action of currently used antiseizure drugs. *Neuropharmacology* 168:107966. 10.1016/j.neuropharm.2020.107966 32120063

[B136] Simeone T. A. Matthews S. A. Samson K. K. Simeone K. A. (2017a). Regulation of brain PPARgamma2 contributes to ketogenic diet anti-seizure efficacy. *Exp. Neurol.* 287 54–64. 10.1016/j.expneurol.2016.08.006 27527983 PMC5110374

[B137] Simeone T. A. Simeone K. A. Rho J. M. (2017b). Ketone bodies as anti-seizure agents. *Neurochem. Res.* 42 2011–2018. 10.1007/s11064-017-2253-5 28397070 PMC5505793

[B138] Sims-Robinson C. Kim B. Rosko A. Feldman E. L. (2010). How does diabetes accelerate Alzheimer disease pathology? *Nat. Rev. Neurol.* 6 551–559. 10.1038/nrneurol.2010.130 20842183 PMC3199576

[B139] Song T. J. Chang Y. Kim A. R. Kim Y. Kim Y. J. (2018). High dietary glycemic load was associated with the presence and burden of cerebral small vessel diseases in acute ischemic stroke patients. *Nutr. Res.* 51 93–101. 10.1016/j.nutres.2017.12.009 29459114

[B140] Spanaki C. Rodopaios N. E. Koulouri A. Pliakas T. Papadopoulou S. K. Vasara E. (2021). The christian orthodox church fasting diet is associated with lower levels of depression and anxiety and a better cognitive performance in middle life. *Nutrients* 13:627. 10.3390/nu13020627 33671993 PMC7919284

[B141] Spence J. D. (2019). Nutrition and risk of stroke. *Nutrients* 11:647. 10.3390/nu11030647 30884883 PMC6470893

[B142] Sullivan P. G. Rippy N. A. Dorenbos K. Concepcion R. C. Agarwal A. K. Rho J. M. (2004). The Ketogenic diet increases mitochondrial uncoupling protein levels and activity. *Ann. Neurol.* 55 576–580. 10.1002/ana.20062 15048898

[B143] Sumathi T. Manivasagam T. Thenmozhi A. J. (2020). “The role of gluten in autism,” in *Personalized Food Intervention and Therapy for Autism Spectrum Disorder Management. Advances in Neurobiology*, Vol. 24 eds Essa M. Qoronfleh M. (Cham: Springer), 469–479. 10.1007/978-3-030-30402-7_14 32006368

[B144] Tan M. S. Yu J. T. Jiang T. Zhu X. C. Tan L. (2013). The NLRP3 inflammasome in Alzheimer’s disease. *Mol. Neurobiol.* 48 875–882. 10.1007/s12035-013-8475-x 23686772

[B145] Tanaka H. Gourley D. D. Dekhtyar M. Haley A. P. (2020). Cognition, brain structure, and brain function in individuals with obesity and related disorders. *Curr. Obesity Rep.* 9 544–549. 10.1007/s13679-020-00412-y 33064270

[B146] Taylor M. K. Swerdlow R. H. Sullivan D. K. (2019). Dietary neuroketotherapeutics for Alzheimer’s disease: an evidence update and the potential role for diet quality. *Nutrients* 11 1–24. 10.3390/nu11081910 31443216 PMC6722814

[B147] Thane P. (2013). The ageing of modern societies: crisis or opportunity? *Historia* 396 333–349.

[B148] Theoharides T. C. Tsilioni I. Patel A. B. Doyle R. (2016). Atopic diseases and inflammation of the brain in the pathogenesis of autism spectrum disorders. *Transl. Psychiatry* 6:e844. 10.1038/tp.2016.77 27351598 PMC4931610

[B149] Thijs R. D. Surges R. O’Brien T. J. Sander J. W. (2019). Epilepsy in adults. *Lancet* 393 689–701. 10.1016/S0140-6736(18)32596-0 30686584

[B150] Tian H. H. Aziz A. R. Png W. Wahid M. F. Yeo D. Png A. L. C. (2011). Effects of fasting during Ramadan month on cognitive function in Muslim athletes. *Asian J. Sports Med.* 2 145–153. 10.5812/asjsm.34753 22375233 PMC3289210

[B151] Tomata Y. Zhang S. Kaiho Y. Tanji F. Sugawara Y. Tsuji I. (2019). Nutritional characteristics of the Japanese diet: a cross-sectional study of the correlation between Japanese Diet Index and nutrient intake among community-based elderly Japanese. *Nutrition* 57 115–121. 10.1016/j.nut.2018.06.011 30157468

[B152] Treiman D. M. (2001). GABAergic mechanisms in epilepsy. *Epilepsia* 42 8–12. 10.1046/j.1528-1157.2001.042Suppl.3008.x 11520315

[B153] Tuttolomondo A. Casuccio A. Buttà C. Pecoraro R. di Raimondo D. della Corte V. (2015). Mediterranean Diet in patients with acute ischemic stroke: relationships between Mediterranean Diet score, diagnostic subtype, and stroke severity index. *Atherosclerosis* 243 260–267. 10.1016/j.atherosclerosis.2015.09.017 26409625

[B154] Uchiki T. Weikel K. A. Jiao W. Shang F. Caceres A. Pawlak D. (2012). Glycation-altered proteolysis as a pathobiologic mechanism that links dietary glycemic index, aging, and age-related disease (in nondiabetics). *Aging Cell* 11 1–13. 10.1111/j.1474-9726.2011.00752.x 21967227 PMC3257376

[B155] Val-Laillet D. Layec S. Guérin S. Meurice P. Malbert C. H. (2011). Changes in brain activity after a diet-induced obesity. *Obesity* 19 749–756. 10.1038/oby.2010.292 21212769

[B156] van de Sande M. M. H. van Buul V. J. Brouns F. J. P. H. (2014). Autism and nutrition: the role of the gut-brain axis. *Nutr. Res. Rev.* 27 199–214. 10.1017/S0954422414000110 25004237

[B157] van Name M. A. Savoye M. Chick J. M. Galuppo B. T. Feldstein A. E. Pierpont B. (2020). A low ω-6 to ω-3 PUFA ratio (n-6:n-3 PUFA) diet to treat fatty liver disease in obese youth. *J. Nutr.* 159 2314–2321. 10.1093/jn/nxaa183 32652034 PMC7467848

[B158] VanItallie T. B. Nonas C. di Rocco A. Boyar K. Hyams K. Heymsfield S. B. (2005). Treatment of Parkinson disease with diet-induced hyperketonemia: a feasibility study. *Neurology* 64 728–730. 10.1212/01.WNL.0000152046.11390.45 15728303

[B159] Vargas D. L. Nascimbene C. Krishnan C. Zimmerman A. W. Pardo C. A. (2005). Neuroglial activation and neuroinflammation in the brain of patients with autism. *Ann. Neurol.* 57 67–81. 10.1002/ana.20315 15546155

[B160] Vergati M. Krasniqi E. Monte G. D. Riondino S. Vallone D. Guadagni F. (2017). Ketogenic diet and other dietary intervention strategies in the treatment of cancer. *Curr. Med. Chem.* 24 1170–1185. 10.2174/0929867324666170116122915 28093985

[B161] Verrotti A. Iapadre G. di Francesco L. Zagaroli L. Farello G. (2020). Diet in the treatment of epilepsy: what we know so far. *Nutrients* 12:2645. 10.3390/nu12092645 32872661 PMC7551815

[B162] Vezzani A. Lang B. Aronica E. (2016). Immunity and inflammation in epilepsy. *Cold Spring Harb. Perspect. Med.* 6 1–22. 10.1101/cshperspect.a022699 26684336 PMC4743070

[B163] Vilić D. (2017). Causes and consequences of increased aging trend in modern society. *Sociol. Discours.* 6 5–34. 10.7251/socen1712005v

[B164] Wheless J. W. (2004). “History and Origin of the Ketogenic Diet,” in *Epilepsy and the Ketogenic Diet*, eds Stafstrom C. E. Rho J. M. (Totowa, NJ: Humana Press), 31–50. 10.1007/978-1-59259-808-3_2

[B165] Williams T. J. Cervenka M. C. (2017). The role for ketogenic diets in epilepsy and status epilepticus in adults. *Clin. Neurophysiol. Pract.* 2 154–160. 10.1016/j.cnp.2017.06.001 30214989 PMC6123874

[B166] Włodarek D. (2019). Role of ketogenic diets in neurodegenerative diseases (Alzheimer’s disease and parkinson’s disease). *Nutrients* 11:169. 10.3390/nu11010169 30650523 PMC6356942

[B167] Woo J. M. Postolache T. T. (2008). The impact of work environment on mood disorders and suicide: evidence and implications. *International Journal on Disability and Human Development* 7 185–200. 10.1515/IJDHD.2008.7.2.185 18836547 PMC2559945

[B168] World Health Organization [WHO] (2003). Diet, nutrition and the prevention of chronic diseases. Report of a Joint WHO/FAO expert consultation. *WHO Tech. Rep. Ser.* 916 1–148. 12768890

[B169] World Health Organization [WHO] (2015). *World Report on Ageing and Health.* Geneva: WHO, 1–260.

[B170] Wyss-Coray T. (2016). Ageing, neurodegeneration and brain rejuvenation. *Nature* 539 180–186. 10.1038/nature20411 27830812 PMC5172605

[B171] Yates K. F. Sweat V. Yau P. L. Turchiano M. M. Convit A. (2012). Impact of metabolic syndrome on cognition and brain: a selected review of the literature. *Arterioscler. Thromb. Vasc. Biol.* 32 2060–2067. 10.1161/ATVBAHA.112.252759 22895667 PMC3442257

[B172] Yehuda S. (2003). Omega-6/omega-3 ratio and brain-related functions. *World Rev. Nutr. Diet.* 92 37–56. 10.1159/000073791 14579682

[B173] Youm Y. H. Nguyen K. Y. Grant R. W. Goldberg E. L. Bodogai M. Kim D. (2015). The ketone metabolite β-hydroxybutyrate blocks NLRP3 inflammasome-mediated inflammatory disease. *Nat. Med.* 21 263–269. 10.1038/nm.3804 25686106 PMC4352123

[B177] Yu D. Zhang X. Shu X. O. Cai H. Li H. Ding D. (2016). Dietary glycemic index, glycemic load, and refined carbohydrates are associated with risk of stroke: a prospective cohort study in urban Chinese women. *Am. J. Clin. Nutrit.* 104, 1345–1351. 10.3945/ajcn.115.129379 27733400 PMC5081713

[B174] Zhou Z. Austin G. Young L. Johnson L. Sun R. (2018). Mitochondrial metabolism in major neurological diseases. *Cells* 7 229–254. 10.3390/cells7120229 30477120 PMC6316877

